# Letter from Italy

**Published:** 1880-09

**Authors:** A. Lagorio

**Affiliations:** Chiavari, Italy


### Foreign Correspondence.

### ARTICLE VIII.

*Messrs. Editors:*—The problem, "What is malaria?" has not only a scientific interest, but, at least for Italy, is also a social problem of the greatest importance. There is no one who does not see, especially in Rome, the truth of this assertion. Great studies regarding this subject have recently been made by Prof. Tomasi Crudeli, of Rome; and here I shall briefly communicate some points of his paper read before the medical society a few months ago. He first occupied himself with the importance of the problem; he noted that the means used to destroy the nascent powers of this morbid agent did not have any other object except to remove one or more of those factors that experience has demonstrated necessary to the development of malaria, as are: 1st, A very elevated temperature; 2d, a persistent humidity of the soil; 3d, the access of air as far as the damp layers of the soil. These methods may limitate or temporarily stop the nascent action of the malarious places, but do not destroy "that particular aptitude of malarious grounds, that constitute the essential difference between them and those grounds, that notwithstanding they have the same composition and may be put in the same exterior condition, are not capable of generating malaria." So he demonstrated how those places where were used the proper beneficial means have been free for many years of malaria, and now were again infected, when these conditions that opposed to its development were ceased. According to the illustrious professor is valued the presumption that the cause of malaria consists in little solid bodies of little specific weight contained in the soil, which are capable of reproducing themselves ad infinitum, when they find all the conditions favorable to their development; that they are living organisms; but to show undoubtedly the parasitic origin of the malarious infection, he admits that necessarily must be proven: 1st, the constant presence of a determined organic species in the various qualities of malarious grounds, and in the air above the same; 2d, that the organic species must be able alone, that is without interferment of any other morbific agent, to generate a true specific intermittent fever. Regarding this object he has made many experiments with Prof. Klebs, of Vienna, and they consist in gathering air and soil of these malarious places, in examining these samples by the microscope, determining the various organic forms that are found, submit this material to liquids to obtain in these the predominance of one of these multiples living elements that existed in the air or in the soil and finally inject these liquids under the skin of rabbits. In this manner they have been able to obtain paroxysms of malarious intermittent fever by the use of one of these liquids containing in great abundance forms of "schizomiceti" pertaining to the gender Bacillus, a form that they consider as the excitant of the malarious infection, and call it "Bacillus malariae." What authorizes the experimenters to attribute the morbigenous action shown by the liquid, to those above described organic forms, is especially the counterproof that taken away by filtering the solid little bodies from the infectant liquid, this looses a great portion of its morbiferous potency. If the results obtained in these researches will be confirmed by new experiments made by the same experimenters and by persons competent as them, the modern experimental pathology can pride itself of one of the greatest discoveries that have been made at our time. I hope that the Prof. Tommaji and Klebs will not abandon this argument of study, and also I hope that if the science will recognize in this microscopical vegetable the ultimate cause of the malarious infection, will know also in future to give us the means for its destruction.

At last during last year has been added to the faculty of medicine, at Genoa, a professorship of dermatology. Not only in Genoa but also in all other universities of Italy the study of skin diseases has stepped forward as one of the leading branches taught. The clinic at Genoa is conducted by Prof. Campana, a very skillful diagnostician and practitioner. Last year the clinic, although in its beginning, has constantly been attended by many patients, and the labors of the professor in working it up have been crowned with the happiest success. In this clinic I have chiefly noted the use and action of the powder of Goa, which is obtained by pounding the woods of various Indian trees, which are known by those natives by the name of Araroba, or Aroriba in their tongue, meaning yellow taint, consisting chiefly in its totality of crysophanic acid, and to this is due its action. The ointment of crysophanic acid is prepared by dissolving it first in benzine and then mixing it with fat in the proportion ten parts to forty of lard; to prevent the erythema and in the face the erysipelas and œdema of the eyelids, Dr. Scarenzio has advised to apply around the limits of the affected part a layer of collodion, so as to circumscribe the erythema; this method very often succeeds, but may also fail. Not wishing to be too tedious in naming all the affections where it was used, I will only say: 1st. That the crysophanic acid is an excellent remedy in psoriasis, where, according to my observations, may prove useful, also in forms more difficult to be cured by this remedy, according to others (psoriasis guttata). 2d. That it is of utility in producing absorption of the papulo—hypertrophic syphiloderma, tubercula hypertrophic; the hypertrophy of the lupoid nodules; also without provoking solution of continuity of the superficies, especially if coajuvated by the precedent abrasion. 3d. That in lepra, produces absorption of the tubercles and of the macula infiltrations. 4th. That it is logic to conclude that this remedy cannot protect against relapses. 5th. That the rapid absorption of the nodules and leprous infiltrations does not facilitate the eruption of new manifestations, nor does it disturb the general condition. 6th. That in lepra may be noted facts of alterated thermogenesis, with relative increase of temperature in the affected part, an increase that sometimes seems to involve also the sound superficies contiguous the same side of the affected territory. The tincture of tayuya has been, during the past years, grandly lauded by some as the great specific in syphilis. In this clinic it had its trials, and I will say more than it ought to have had; no good effects could be gotten and was finally banished from the clinic as the remedy in syphilis. The tayuya did not at all respond to the clamor made by somebody. Not only is it behind, according to Dr. Geber, a thousand miles to the potassic iodide and mercury, but is inferior to those syphilitic remedies that the physicians of our day make use of, because of middling efficacy.

I must refer before I close to a pathognomonic symptom, very little known in the fracture of the neck of the femur. It is a traditional practice in this hospital, to explore, whenever the fracture is suspected, the small space that is found between the trochanter and the crest of the ilium. In placing the two extremities in the same position, instead of the considerable resistance given in the sound limb by the tension of the extensor muscle of the fascia lata, and of the gluteus medius, a deep hollowness is found in the affected limb, due certainly to the diminished tension of the above named muscles by the approaches of their points of attachment. A. LAGORIO, M. D.

CHIAVARI, Italy, July 14, 1880.

---

ETIOLOGY AND PROPHYLAXIS OF PREMATURE BALDNESS. (*Revue Méd.* No. 22, 1880.)

According to Dr. Ellinger water and the regular use of ablutions of the scalp are the principal factors of premature baldness. In 100 individuals troubled with alopecia 85 used ablutions of the head since early youth; and among those who had preserved a luxuriant head of hair to an advanced age eight per cent. only had this habit. At the point of implantation of the hairs, the water forms with the epidermic layers and the sebaceous matter of the hair, an emulsion, an oil which concretes and obstructs the hair follicles which lead first to their distension, then to their atrophy.

M. Ellinger thinks a well performed mechanical cleansing, with sand or a wire brush, is not only the most sure means of prevention, but even a good curative for the fall of hair at the commencement.

